# Early Latin American experience with faricimab for retinal diseases: FARI-LATAM study for the Pan-American collaborative retina study (PACORES) group

**DOI:** 10.1186/s40942-026-00804-7

**Published:** 2026-02-21

**Authors:** Lihteh Wu, Sergio Rojas, Xi Rao, Michael Politis, José Antonio Roca, Marcio Nehemy, Maria H. Berrocal, Jans Fromow-Guerra, Karen Barraza-Lino, Carlos Lopez, Mauricio Maia, Andrea Cardenas-Mirón, Gregorio Gabela, Olivia Baldivieso-Hurtado, Cristina Gabela, Lisseth Chinchilla, Juan Ignacio Verdaguer, Marcelo Zas, Melissa Young, Gerardo García-Aguirre, J. Abel Ramirez-Estudillo, Nassim Abreu, Miriam Flores, Wandsy Velez, Yazmin Baez, Andrea Arriola-Lopez, Antonio Marcelo Casella

**Affiliations:** 1 Asociados de Macula Vitreo y Retina de Costa Rica, San Jose, Costa Rica; 2 Department of Ophthalmology, School of Medicine, Illinois Eye and Ear Infirmary, University of Illinois Chicago, Chicago, USA; 3 Centro Oftalmológico Puerta Clara Cuernavaca Morelos, Mexico City, México; 4 Departamento de Retina, Hospital de la Luz, Mexico City, Mexico; 5 XR Oftalmologia, Santiago, Chile; 6 Panama Eye Center, Panama City, Panama; 7 Oftalmólogos Contreras, Instituto de Ojos Primavera, Lima, Peru; 8 Federal University of Minas Gerais School of the Medicine, Belo Horizonte, Brazil; 9 Drs Berrocal and Associates, San Juan, Puerto Rico; 10 Asociacion para Evitar la Ceguera en Mexico, Hospital Luis Sanchez Bulnes, Mexico City, Mexico; 11 Departamento de Oftalmología, Clínica Internacional, Lima, Peru; 12 Centro Panamericano de Ojos, San Salvador, El Salvador; 13 Vitreoretinal Service, Universidade Federal de Sao Paulo, Sao Paulo, Brasil; 14 Vitreoretinal Service Hospital de Olhos Participações SA, Assis/Presidente Prudente, SP São Paulo, Brasil; 15 Departamento de Retina y Vítreo, Clínica Visualiza, Ciudad de Guatemala, Guatemala; 16 Centro de la Visión, Hospital Metropolitano, Quito, Ecuador; 17 Clinica de Ojos Baldivieso, Santa Cruz, Bolivia; 18 Pharmacology Department, Universidad de los Andes Núcleo Táchira, Táchira, Venezuela; 19 Fundación Oftalmológica Los Andes, Universidad de los Andes, Santiago, Chile; 20 Catedra de Oftalmología, Hospital de Clinicas Jose de San Martín, Universidad de Buenos Aires, Buenos Aires, Argentina; 21 Oftalmo Advanced Clinic, Panama City, Panamá; 22 School of Medicine and Health Sciences, Tecnologico de Monterrey, Mexico City, Mexico; 23 Centro Oftalmologico Mira, México City, Mexico; 24 Dr Elías Santana Hospital, Santo Domingo, Dominican Republic; 25 Departamento Oftalmologia, Hospital Militar Central, San Salvador, El Salvador; 26 Retina Associates, Guaynabo, Puerto Rico; 27 Innovación Ocular, Guatemala City, Guatemala; 28 Londrina State University, Londrina, Brazil

**Keywords:** Age-related macular degeneration, AMD, Diabetic macular edema, DME, Retinal vein occlusion, RVO, Faricimab, Angiopoietins, VEGF

## Abstract

**Purpose:**

To report the early visual outcomes of eyes with a variety of retinal conditions that were treated with intravitreal faricimab during routine clinical practice in Latin America.

**Methods:**

Retrospective multicenter case series of 757 eyes of 732 patients who underwent at least one intravitreal injection of faricimab and had a least 4 weeks of follow-up.

**Results:**

Our cohort consisted of 392 eyes with neovascular age-related macular degeneration (NV-AMD) (naive = 105; prior tx = 287); 225 with DME (naive = 74; prior tx = 151); 50 with central retinal vein occlusion (CRVO) (naive = 29; prior tx = 21); 20 with previously treated branch retinal vein occlusion (BRVO) and 69 (naive = 25; prior tx = 44) with other conditions. After a mean follow-up of 26.6 weeks and 4 injections, eyes with treatment naive NV-AMD eyes gained 9.1 letters (*p* < 0.0001) with a last mean treatment interval of 9.5 weeks. Previously-treated NV-AMD eyes gained 6.6 to 9.5 letters (*p* = 0.00146 and *p* < 0.0001) after a mean follow-up of 29 to 42 weeks and 3.8 to 4.3 injections with a last mean treatment interval of 9.2 to 12.1 weeks. In treatment naive diabetic macular edema (DME) eyes, after a mean follow-up of 24.8 weeks and 4.2 injections, there was a gain of 12 letters (*p* < 0.0001) with a last mean treatment interval of 9.2 weeks. In previously-treated DME eyes after a mean follow-up of 27.2 to 44.2 weeks and 3.8 to 3.9 to injections, there was a gain of 6.1 to 11.7 letters (*p* < 0.0001) with a last mean treatment interval of 9.6 to 15.2 weeks. Treatment-naive CRVO eyes gained 22.6 letters (*p* = 0.00369) after a mean follow-up of 26.2 weeks and 3.7 injections. Previously treated CRVO eyes gained 9.8 letters (0.5233) after a mean follow-up of 21.8 weeks and 3.4 injections. Previously-treated BRVO eyes gained 5.4 letters (*p* = 0.00862) after a mean follow-up of 28.1 weeks and 3.5 injections and a last treatment interval of 11.1 weeks. A total of 38 eyes discontinued treatment with faricimab and switched back to aflibercept. There were 3 cases of anterior uveitis that were treated with topical steroids. None of the eyes developed infectious endophthalmitis or occlusive retinal vasculitis.

**Conclusions:**

The early Latin American experience with faricimab is promising. On average treated eyes had functional and anatomic gains from baseline.

## Introduction

Several diseases of the posterior segment of the eye, including diabetic macular edema (DME), retinal vein occlusions (RVO) and age-related macular degeneration (AMD) are leading causes of blindness and visual disability worldwide. These conditions are often characterized by cystoid macular edema (CME), retinal neovascularization (RNV), macular neovascularization (MNV) and choroidal neovascularization (CNV). Vascular endothelial growth factor (VEGF) plays an important role in the pathobiology of these conditions [1]. Intravitreal injections of anti-VEGF agents are currently the treatment of choice and since their introduction, blindness rates secondary to these diseases have fallen importantly [2]. Nevertheless patients under anti-VEGF treatment need to be typically seen and injected every 1 to 4 months depending on the response to treatment. The frequency of these visits and injections constitutes a major burden and may influence treatment compliance and lead to worse outcomes than those obtained in randomized clinical trials. Therefore despite the benefits of anti-VEGF treatments, the treatment is burdensome and the results from routine clinical practice fall short of the results obtained in clinical trials. Vision threatening complications such as endophthalmitis and retinal detachment although rare may still occur. Repeated injections increase the risk of these complications [3–7]. 

It comes as no surprise that researchers are constantly evaluating new therapeutic pathways for these conditions. One of these pathways is the angiopoietin–Tie signaling pathway. The angiopoietins are gatekeepers of vascular homeostasis and key drivers of vascular remodeling and maturation. Many have recognized the value of targeting the Ang/Tie signaling as a therapeutic target [8]. 

Faricimab-svoa is a heterodimeric bispecific monoclonal antibody (F. Hoffmann-La Roche, Basel, Switzerland) that targets both VEGF-A and Ang2 [9]. Several pivotal trials demonstrated that the BCVA gains with faricimab were non-inferior to those obtained by 2 mg of aflibercept in NV-AMD, DME and RVO. Furthermore these results were obtained with less injections of faricimab suggesting a longer duration of action as compared to 2 mg of aflibercept [10–12]. Based on these results, faricimab was approved by regulatory agencies for NV-AMD, DME and RVO.

Since real world data in Latin American populations remain limited, we wanted to report the early visual and anatomic outcomes of eyes with a variety of retinal conditions that were treated with intravitreal faricimab during routine clinical practice in Latin America.

## Methods

### Study design and data collection

The tenets of the Declaration of Helsinki were adhered to. Institutional review board approval was obtained at each participating center, if specifically required for a retrospective study (Cesar Milstein Hospital Buenos Aires, Argentina; Hospital Militar Central San Salvador El Salvador; Clinica Ricardo Palma Lima Peru). We conducted a multicenter retrospective study of 757 eyes of 732 patients with a variety of retinal conditions that were treated with intravitreal faricimab in Latin America. We reviewed the clinical records of these patients by circulating a standardised anonymized data collection template that was designed to capture demographic information, treatment history, follow up period, spectral domain optical coherence tomography (SD-OCT) results and visual acuity at different time points. Each investigator was responsible for entering patient specific details into the shared Excel-based template.

### Patient eligibility and exclusion criteria

All patients who underwent at least one intravitreal injection of faricimab and had a baseline BCVA, baseline macular SD-OCT and at least 4 weeks of follow-up with BCVA and macular SD-OCT were included.

### Examination and treatment procedures

At baseline each patient underwent a complete ophthalmic examination and SD-OCT (Spectralis, Heidelberg Engineering, Heidelberg, Germany; Cirrus, Carl Zeiss Meditec, Dublin, CA, USA; iVue OCT and Optovue Solix, Optovue, Fremont, CA, USA; Topcon Maestro, Yamagata, Japan). A volume scan centered on the fovea was performed. The central subfield thickness (CST) and the total macular volume (TMV) were calculated automatically by each machine using their proprietary algorithms. The scans were reviewed and manual corrections were performed in case of segmentation errors. Follow-up scans were performed with the same OCT machine and the same volume scan as the baseline scans. Other imaging modalities were done at the discretion of the treating physician.

Patients were injected with 6 mg of faricimab through the pars plana after standard preparation with topical anesthesia and 5% povidone iodine. Patients were seen and treated at the discretion of the treating physician. Repeat SD-OCT were also repeated at the discretion of the treating physician.

### Statistical analysis

Conversion of BCVA to a logMAR scale was performed to facilitate statistical analysis of the data. Statistical analysis was performed using GraphPad Prism ^®^ (Version 8.0 for the Macintosh OSX, GraphPad Software, San Diego, California, USA). Continuous variables were tested for normality using the Shapiro-Wilk test. Normally distributed data are expressed as a mean with 95% confidence intervals (CI). One way repeated measures ANOVA and the Friedman test for repeated measures were calculated accordingly to compare means according to the number of injections. A p value of < 0.05 was considered significant.

The main outcome measured was the change in BCVA from baseline to last follow-up. Secondary outcomes measured included change from baseline in CST, TMV and total number of injections at last follow-up. The analyses were based on available-case data per visit, no imputation was used for missing data and the same eyes contributed to all time points where individual data was available. The “last visit” includes all available eyes irrespective of intermediate visit participation.

## Results

The results are presented in several tables according to indication and to prior treatment. As an ongoing real world experience study, all patients have a different follow-up time. Therefore the number of patients (n) will vary at different cut-off points (i.e. number of injections).

### Baseline characteristics

A total of 757 eyes fulfilled the inclusion and exclusion criteria. Indications for faricimab injection included NV-AMD, DME, CME secondary to central retinal vein occlusion (CRVO), CME secondary to branch retinal vein occlusion (BRVO) plus several other retinal conditions including myopic CNV, polypoidal choroidal vasculopathy (PCV), pachychoroid neovasculopathy, CNV secondary to central serous chorioretinopathy (CSCR), proliferative diabetic retinopathy (PDR), idiopathic CNV, CME secondary to macroaneurysm, CME secondary to retinitis pigmentosa and pseudophakic CME. There were 233 treatment naive eyes compared to 524 previously treated eyes.

Eyes were sub-divided into treatment naive and previously treated eyes according to indication (i.e. NV-AMD, DME, CRVO, BRVO and others). The previously treated eyes were further subdivided into those that were not doing well and those that were seeking a treatment interval extension.

### NV-AMD

A total of 392 eyes were treated for NV-AMD. Of these, 105 were treatment naive and 287 were previously treated with other anti-VEGF drugs. Of the previously treated eyes, 68 eyes were switched to try to increase the treatment interval and the remaining 219 were switched due to a poor response with the prior treatment.

### Treatment Naive NV-AMD

The average age of the treatment naive patients was 78.4 (95% CI: 76.4 to 80.4) years of age. Patients had a mean total follow-up of 26.6 (95% CI: 21.8 to 31.4) weeks. During this time period, patients received a mean of 4 (95% CI: 3.5 to 4.5) faricimab injections gaining a mean of 9.1 letters (*p* < 0.0001, Friedman test for repeated measures) with a last treatment interval of 9.5 weeks. Table 1 summarizes the visual, anatomic outcomes and the time interval between injections following the first 4 injections and at the last visit in the treatment naive NV-AMD eyes. None of the eyes were switched to an alternate drug.

**Table 1:** Treatment Naive eyes with neovascular Age-Related macular degeneration

	Baseline	1 Injection	2 Injections	3 Injections	4 Injections	Last Visit	*P*
BCVA logMAR (Snellen) 95% C.I.	0.86 (20/145) (0.76 to 0.97)	0.72 (20/105) (0.61 to 0.83)	0.75 (20/112) (0.61 to 0.89)	0.73 (20/107) (0.58 to 0.88)	0.63 (20/85) (0.50 to 0.76)	0.68 (20/96) (0.57 to 0.79)	*P* < 0.0001
Cumulative Letter Gain 95% C.I.		6.1 (3.7 to 8.4)	6.1 (2.6 to 9.6)	7.6 (2.9 to 12.2)	11 (6.5 to 25.2)	9.1 (5.6 to 12.6)	*P* < 0.0001
CST (µm) 95% C.I.	417 (371 to 463)	274 (244 to 305)	261 (231 to 291)	272 (245 to 298)	248 (220 to 277)	267 (241 to 293)	*P* < 0.0001
TMV (mm^3^) 95% C.I.	9.53 (8.92 to 10.14)	8.18 (7.69 to 8.66)	7.85 (7.38 to 8.32)	7.87 (7.39 to 8.35)	7.79 (7.19 to 8.39)	8.07 (7.57 to 8.58)	*P* < 0.0001
Time Interval Between Injections (weeks) 95% C.I.		5.6 (4.8 to 6.3) 2	6.5 (5.5 to 7.5) 4	7.6 (6.5 to 8.8) 8	11.2 (9.2 to 13.3) 10	9.5 (8.1 to 10.9) 8	*P* < 0.0001
n	105	105	63	63	47	105	

BCVA = best corrected visual acuity; CI = Confidence interval; CST = central subfield thickness; TMV = total macular volume

### Previously treated NV-AMD

The average age of the 68 previously treated patients who were switched to try to increase the treatment interval was 79.1 (95% CI:77.6 to 81.4) years of age. Prior to the first faricimab injection, these eyes received a mean of 14.5 (95% CI: 11 to 18) injections including bevacizumab, ranibizumab, aflibercept and brolucizumab. The average interval between the last anti-VEGF and the first faricimab injection was 15.1 (95% CI: 10 to 20.3) weeks. Patients had a mean total follow-up of 41.7 (95% CI: 35.2 to 48.3) weeks. During this time, patients received a mean of 4.3 (95% CI: 3.7 to 4.9) faricimab injections gaining a mean of 6.6 letters (*p* = 0.00146, Friedman test for repeated measures). The last treatment interval was reported to be of 12.1 weeks. Table 2 summarizes the visual, anatomic outcomes and the time interval between injections following the first 4 injections and at the last visit in the previously treated NV-AMD eyes that were switched for a treatment interval extension. Of these 68 eyes, one eye was switched back to bevacizumab after the fourth faricimab injection. Four eyes were switched back to aflibercept. One eye was switched back after the first faricimab, another after the second, another after the third and the last one after the seventh injection.

**Table 2:** Previously treated eyes with neovascular Age-Related macular degeneration switched to faricimab to extend the treatment interval

	Baseline	1 Injection	2 Injections	3 Injections	4 Injections	Last Visit	*P*
BCVA logMAR (Snellen) 95% C.I.	0.66 (20/91) 0.52 to 0.81	0.57 (20/74) (0.43 to 0.71)	0.51 (20/65) (0.36 to 0.67)	0.49 (20/62) (0.33 to 0.65)	0.38 (20/48) (0.25 to 0.51)	0.53 (20/68) (0.41 to 0.65)	*p* = 0.00146
Cumulative Letter Gain 95% C.I.		5.0 (1.5 to 8.5)	8.1 (2.3 to 13.9)	4.6 (-1.4 to 10.4)	8.5 (1.2 to 15.8)	6.6 (1.4 to 11.8)	*p* = 0.00146
CST (µm) 95% C.I.	304 (274 to 335)	266 (237 to 295)	272 (238 to 306)	252 (219 to 286)	268 (222 to 314)	253 (227 to 279)	*p* = 0.00003
TMV (mm^3^) 95% C.I.	7.98 (7.48 to 8.48)	7.62 (7.14 to 8.11)	7.75 (7.24 to 8.26)	7.49 (6.95 to 8.03)	7.37 (6.78 to 7.97)	7.54 (7.09 to 7.98)	*p* = 0.00009
Time Interval Between Injections (weeks) 95% C.I.	15.1 (10 to 20.3)	8.7 (7.4 to 10)	9.4 (8.1 to 10.6)	11.7 (9.8 to 13.6)	11.5 (9.7 to 13.3)	12.1 (10.4 to 13.7)	*p* = 0.02
n	68	68	52	46	37	68	

BCVA = best corrected visual acuity; CI = Confidence interval; CST = central subfield thickness; TMV = total macular volume

The average age of the 219 previously treated patients with worsening NV-AMD who were switched to faricimab was 77.5 (95% CI: 76.3 to 78.7) years of age. On average these patients received a mean of 12.3 (95% CI: 10.4 to 14.2) prior injections of an anti-VEGF including bevacizumab, ranibizumab, aflibercept and brolucizumab. The average interval between the last anti-VEGF and the first faricimab injection was 15.7 (95% CI: 10 to 21.4) weeks. Patients had a mean total follow-up of 28.6 (95% CI: 25.8 to 31.5) weeks and received a mean of 3.8 (95% CI: 3.4 to 4.2) faricimab injections. During this time period eyes gained an average of 9.5 letters (*p* < 0.0001, Friedman test for repeated measures) with a last treatment interval of 9.2 weeks. Table 3 summarizes the visual, anatomic outcomes and the time interval between injections following the first 4 injections and at the last visit in the previously treated eyes switched to faricimab because of a poor response. Of these 219 eyes, there were 19 eyes that were switched back to aflibercept and one was switched back to bevacizumab. The eye that was switched back to bevacizumab was switched back after the first faricimab injection. One eye was switched to aflibercept after the first faricimab injection, 8 eyes after the second injection, 3 eyes after the third injection, 2 eyes after the fourth injection, 2 eyes after the fifth injection and 2 eyes after the sixth injection.

### DME

**Table 3:** Previously treated eyes with worsening neovascular Age-Related macular degeneration that were switched to faricimab

	Baseline	1 Injection	2 Injections	3 Injections	4 Injections	Last Visit	*P*
BCVA logMAR (Snellen) 95% C.I.	0.69 (20/98) (0.62 to 0.76)	0.61 (20/81) (0.54 to 0.67)	0.53 (20/68) (0.46 to 0.60)	0.51 (20/65) (0.43 to 0.58)	0.49 (20/62) (0.41 to 0.58)	0.53 (20/68) (0.47 to 0.59)	*P* < 0.0001
Cumulative Letter Gain 95% C.I.		4.5 (2.7 to 6.4)	7.8 (5.1 to 10.6)	10 (6.6 to 13.3)	11.6 (7.1 to 16.1)	9.5 (7 to 12.1)	*P* < 0.0001
CST (µm) 95% C.I.	369 (348 to 390)	303 (285 to 322)	308 (287 to 330)	305 (280 to 330)	293 (262 to 323)	281 (264 to 298)	*P* < 0.0001
TMV (mm^3^) 95% C.I.	8.6 (8.26 to 8.94)	7.72 (7.48 to 7.95)	7.81 (7.52 to 8.09)	7.95 (7.51 to 8.39)	8.11 (7.70 to 8.52)	7.68 (7.36 to 8)	*P* < 0.0001
Time Interval Between Injections (weeks) 95% C.I.	15.7 (10 to 21.3)	8.1 (7.3 to 8.8)	8.6 (7.8 to 9.4)	9.2 (8.3 to 10.2)	8.89 (7.8 to 10)	9.2 (8.4 to 10)	*p* = 0.3467
n	219	219	148	121	83	219	

BCVA = best corrected visual acuity; CI = Confidence interval; CST = central subfield thickness; TMV = total macular volume

The BCVA gains and losses of all NV-AMD eyes stratified by the number of injections are summarized in Table 4.

**Table 4:** Gain and loss of BCVA lines in NV-AMD

	Gain > 2 lines	Gain or Losses Within 2 Lines	Loss > 2 lines
Naive After 1 Injection	40/105 (38.1%)	62/105 (59%)	3/105 (2.9%)
Naive After 2 Injections	20/63 (31.7%)	40/63 (63.5%)	3/63 (4.8%)
Naive After 3 Injections	28/63 (44.4%)	30/63 (47.6%)	5/63 (7.9%)
Naive After 4 Injections	28/47 (59.6%)	15/47 (31.9%)	4/47 (8.5%)
Naive at the Last follow-up	46/105 (43.8%)	54/105 (51.4%)	5/105 (4.8%)
Prior Tx to Increase Tx Interval After 1 Injection	17/68 (25%)	47/68 (69.1%)	4/68 (5.9%)
Prior Tx to Increase Tx Interval After 2 Injections	18/52 (34.6%)	30/52 (57.7%)	4/52 (7.7%)
Prior Tx to Increase Tx Interval After 3 Injection	10/46 (21.7%)	32/46 (69.6%)	4/46 (8.7%)
Prior Tx to Increase Tx Interval After 4 Injections	13/37 (35.1%)	20/37 (54%)	4/37 (10.8%)
Prior Tx to Increase Tx Interval at the Last Follow-Up	21/68 (30.9%)	39/68 (57.4%)	8/68 (11.8%)
Prior Tx Due to Worsening After 1 Injection	79/219 (36.1%)	126/219 (57.5%)	14/219 (6.4%)
Prior Tx Due to Worsening After 2 Injections	62/148 (41.9%)	74/148 (50%)	12/148 (8.1%)
Prior Tx Due to Worsening After 3 Injections	53/121 (43.8%)	55/121 (45.4%)	13/121 (10.7%)
Prior Tx Due to Worsening After 4 Injections	39/83 (47%)	35/83 (42.2%)	9/83 (10.8%)
Prior Tx Due to Worsening at the last Follow-Up	89/219 (40.6%)	110/219 (50.2%)	20/219 (9.2%)

BCVA: best corrected visual acuity; NV-AMD: neovascular age-related macular degeneration; Tx: treatment

### DME

A total of 225 eyes were treated for DME. Of these, 74 were treatment naive and 151 eyes were previously treated with other anti-VEGF drugs including bevacizumab, ranibizumab, aflibercept and brolucizumab. Of the previously treated eyes, 18 eyes were switched to try to increase the treatment interval and the remaining 133 were switched due to a poor response with the prior treatment.

### Treatment Naive DME

The average age of the treatment naive patients was 63.1 (95% CI: 61.3–66.3) years of age. Patients had a mean total follow-up of 24.8 (95% CI: 20.1 to 29.5) weeks during which they received a mean of 4.2 (95% CI: 3.5 to 4.9) injections and gained a mean of 12 letters (*p* < 0.0001, Friedman test for repeated measures) over this time period. The last treatment interval was of 9.2 weeks. Table 5 summarizes the visual, anatomic outcomes and the time interval between injections following the first 4 injections and at the last visit in the treatment naive DME eyes. In addition outcomes at the last visit were also included. None of the eyes were switched to an alternate treatment.

**Table 5:** Outcomes of eyes with treatment Naive diabetic macular edema

	Baseline	1 Injection	2 Injections	3 Injections	4 Injections	Last Follow-Up	*P* value *
BCVA logMAR (Snellen) 95% C.I.	0.62 (20/83) (0.52–0.72)	0.52 (20/66) (0.42–0.61)	0.44 (20/55) (0.33–0.56)	0.49 (20/62) (0.36–0.62)	0.51 (20/65) (0.39–0.63)	0.38 (20/48) (0.32 to 0.45)	*p* < 0.0001
Cumulative Letter Gain 95% C.I.		6.4 (2.9 to 9.9)	12.2 (7.1 to 17.3)	13.2 (6.4 to 20.1)	13.5 (5.2 to 21.8)	12 (7.4 to 16.7)	*p* < 0.0001
CST (µm) 95% C.I.	373 (341–404)	292 (263–320)	269 (245–292)	255 (235–276)	241 (223–258)	255 (240 to 269)	*p* < 0.0001
TMV (mm^3^) 95% C.I.	9.02 (8.29 to 9.75)	7.94 (7.19 to 8.68)	7.3 (6.42 to 8.18)	7.26 (6.38 to 8.13)	6.78 (5.67 to 7.89)	7.69 (7.11 to 8.28)	*p* < 0.0001
Time Interval Between Injections (Weeks) 95% C.I.		6.8 (5.3–8.2)	6.1 (5-7.3)	8 (6-9.9)	9.7 (6.2–13.1)	9.2 (7.3 to 11.1)	*p* < 0.0001
n	74	74	44	44	35	74	

BCVA = best corrected visual acuity; CI = confidence interval; * Friedman test for repeated measures

### Previously treated DME

The average age of the previously treated patients that were switched to try to increase the treatment interval was 68.4 (CI 95%: 63.2 to 73.6) years of age. Prior to the first faricimab injection, these eyes received a mean of 11.7 (95% CI: 6.8 to 16.6) injections including bevacizumab, ranibizumab, aflibercept, dexamethasone implant and brolucizumab. The average interval between the last anti-VEGF and the first faricimab injection was 14.7 (95% CI: 7.7 to 21.8) weeks. Patients had a mean total follow-up of 44.2 (95% CI: 32.4 to 56.1) weeks. During this time, patients received a mean of 3.8 (95% CI: 2.9 to 4.8) faricimab injections and had a mean gain of 11.7 letters (*p* < 0.0001 Friedman test for repeated measures). The last treatment interval was of 15.2 weeks. Table 6 summarizes the visual, anatomic outcomes and the time interval between injections following the first 4 injections and at the last visit in the previously treated DME eyes that were switched for a treatment interval extension. None of these 18 eyes were switched to an alternative drug.

**Table 6:** Previously treated eyes with diabetic macular edema switched to faricimab to extend the treatment interval

	Baseline	1 Injection	2 Injections	3 Injections	4 Injections	Last Visit	*p*
BCVA logMAR (Snellen) 95% C.I.	0.57 (20/74) (0.41 to 0.74)	0.39 (20/49) (0.23 to 0.54)	0.36 (20/46) (0.15 to 0.56)	0.43 (20/54) (0.18 to 0.69)	0.48 (20/60) (0.08 to 0.87)	0.34 (20/44) (0.16 to 0.52)	*p* < 0.0001
Cumulative Letter Gain 95% C.I.		9.1 3.7 to 14.5	12.8 5 to 20.8	10.2 2.2 to 18.2	15 0.7 to 29.3	11.7 5.3 to 18.2	*p* < 0.0001
CST (µm) 95% C.I.	272 (227 to 317)	239 (199 to 279)	219 (185 to 253)	234 (213 to 255)	217 (188 to 246)	225 (190 to 261)	*p* < 0.0001
TMV (mm^3^) 95% C.I.	9.2 (8.54 to 9.86)	8.84 (8.28 to 9.40)	8.43 (7.81 to 9.06)	8.8 ( 8.14 to 9.46)	8.65 (8.24 to 9.07)	8.8 (8.26 to 9.33)	*p* < 0.0001
Time Interval Between Injections weeks 95% C.I.	14.7 (7.7 to 21.8)	11.8 (4.9 to 18.8)	10.1 (6.4 to 13.8)	13.2 (8.8 to 17.6)	13.1 (9.1 to 17.1)	15.2 (11.9 to 18.6)	*p* < 0.0001
n	18	18	16	13	8	18	

BCVA = best corrected visual acuity; CI = confidence interval; p value Friedman Test for Repeated Measures

The average age of the 133 previously treated patients that were switched because of a poor response with the prior drug was 64.9 (95% CI: 63.2 to 66.6) years of age. Prior to the first faricimab injection, these eyes received a mean of 7.4 (95% CI: 6.2 to 8.6) injections including bevacizumab, ranibizumab, aflibercept, dexamethasone implant and brolucizumab. The average interval between the last anti-VEGF and the first faricimab injection was 13.9 (95% CI: 11.3 to 16.4) weeks. Patients had a mean total follow-up of 27.2 (95% CI: 22.9 to 31.5) weeks. During this time, patients received a mean of 3.9 (95% CI: 3.5 to 4.3) faricimab injections and had a mean gain of 6.1 letters (*p* < 0.0001, Friedman test for repeated measures). The last reported treatment interval was of 9.6 weeks. Table 7 summarizes the visual, anatomic outcomes and the time interval between injections following the first 4 injections and at the last visit in the previously treated DME eyes that were switched because of a poor response. Of these 133 eyes, there were 4 eyes that were switched back to aflibercept. One switched after the first faricimab injection, two after the second faricimab injection and one switched after the fourth faricimab injection. The BCVA gains and losses of all DME eyes stratified by the number of injections are summarized in Table 8.

**Table 7:** Previously treated eyes with worsening diabetic macular edema that were switched to faricimab

	Baseline	1 Injection	2 Injections	3 Injections	4 Injections	Last Visit	*P*
BCVA logMAR (Snellen) 95% C.I.	0.70 (20/101) 0.63 to 0.78	0.61 (20/81) 0.54 to 0.68	0.54 (20/69) 0.44 to 0.63	0.55 (20/71) 0.46 to 0.64	0.48 (20/61) 0.40 to 0.57	0.58 (20/76) 0.51 to 0.65	*p* < 0.0001
Cumulative Letter Gain 95% C.I.		4.9 (2.5 to 7.3)	8.2 (5 to 11.5)	7.6 (3.8 to 11.5)	10.3 (5.1 to 15.6)	6.1 (3.5 to 8.7)	*p* < 0.0001
CST (µm) 95% C.I.	431 (402 to 459)	377 (331 to 423)	342 (308 to 377)	334 (302 to 368)	308 (285 to 331)	318 (293 to 342)	*p* < 0.0001
TMV (mm^3^) 95% C.I.	8.91 (8.38 to 9.43)	8.2 (7.62 to 8.77)	7.52 (6.78 to 8.27)	7.32 (6.62 to 8.02)	7.03 (6.63 to 7.73)	7.88 (7.37 to 8.40)	*p* < 0.0001
Time Interval Between Injections (weeks) 95% C.I.	13.9 (11.3 to 16.4)	6.9 (5.9 to 7.9)	7.8 (5.9 to 9.8)	8 (6.6 to 9.5)	11.7 (8.9 to 14.4)	9.6 (7.9 to 11.4)	*p* < 0.0001
n	132	132	110	70	60	132	

BCVA = best corrected visual acuity; C.I.= confidence interval; p value Friedman Test for Repeated Measures

**Table 8:** Gain and loss of BCVA lines in diabetic macular edema

	Gain > 2 lines	Gain or Losses Within 2 Lines	Loss > 2 lines
Naive After 1 Injection	26/74 (35.1%)	44/74 (59.4%)	4/74 (5.4%)
Naive After 2 Injections	21/44 (47.7%)	22/44 (50%)	1/44 (2.3%)
Naive After 3 Injections	21/44 (47.7%)	19/44 (43.2%)	4/44 (9.1%)
Naive After 4 Injections	15/35 (42.8%)	19/35 (54.3%)	1/35 (2.9%)
Naive at the Last follow-up	31/74 (41.9%)	40/74 (54%)	3/74 (4.1%)
Prior Tx to Increase Tx Interval After 1 Injection	9/18 (50%)	8/18 (44.4%)	1/18 (5.6%)
Prior Tx to Increase Tx Interval After 2 Injections	9/16 (56.2%)	6/16 (37.5%)	1/16 (6.3%)
Prior Tx to Increase Tx Interval After 3 Injection	7/13 (53.8%)	5/13 (38.5%)	1/13 (7.7%)
Prior Tx to Increase Tx Interval After 4 Injections	5/8 (62.5%)	2/8 (25%)	1/8 (12.5%)
Prior Tx to Increase Tx Interval at the Last Follow-Up	10/18 (55.5%)	7/18 (38.9%)	1/18 (5.6%)
Prior Tx Due to Worsening After 1 Injection	46/133 (34.6%)	78/133 (58.6%)	9/133 (6.8%)
Prior Tx Due to Worsening After 2 Injections	42/110 (38.2%)	65/110 (59.1%)	3/110 (2.7%)
Prior Tx Due to Worsening After 3 Injections	23/70 (32.9%)	44/70 (62.8%)	3/70 (4.3%)
Prior Tx Due to Worsening After 4 Injections	23/60 (38.3%)	30/60 (50%)	7/60 (11.7%)
Prior Tx Due to Worsening at the last Follow-Up	46/133 (34.6%)	77/133 (57.9%)	10/133 (7.5%)

BCVA: best corrected visual acuity; Tx: treatment

### CRVO

A total of 50 eyes were treated for CRVO. Of these, 29 eyes were treatment naive and 21 eyes were previously treated with other anti-VEGF drugs. All 21 eyes with a prior treatment were switched due to a poor response with the prior treatment.

### Treatment Naive CRVO

The average age of the treatment naive patients was 71.8 (95% CI: 65.9 to 77.7) years of age. Patients had a mean total follow-up of 26.2 (95% CI: 15.6 to 36.9) weeks. During this time, patients gained a mean of 22.6 letters (*p* = 0.00369 Friedman test for repeated measures) after receiving a mean of 3.7 (95% CI: 2.6 to 4.8) injections over this time period. The last treatment interval was of 8.8 weeks. Table 9 summarizes the visual, anatomic outcomes and the time interval between injections in the treatment naive CRVO eyes following 3 intravitreal faricimab injections and at the last follow-up. None of these eyes were switched to an alternative drug.

**Table 9:** Eyes with treatment Naive central retinal vein occlusion

	Baseline	1 Injection	2 Injections	3 Injections	Last Visit	*P*
BCVA logMAR (Snellen) 95% C.I.	1.31 (20/407) 1.08 to 1.54	1.12 (20/266) 0.85 to 1.40	1.17 (20/297) 0.75 to 1.60	0.86 (20/145) 0.45 to 1.27	0.83 (20/136) 0.60 to 1.07	*p* = 0.00369
Cumulative Letter Gain 95% C.I.		12.7 -2.7 to 28	14.4 -2.4 to 31.2	22.8 3.3 to 43.2	22.6 9.3 to 36	*p* = 0.00369
CST (µm) 95% C.I.	491 410 to 572	319 269 to 370	269 235 to 304	328 160 to 495	287 229 to 344	*p* = 0.0001
TMV (mm3) 95% C.I.	11.08 8.12 to 10.39	9.25 8.12 to 10.4	8.43 6.70 to 10.16	8.37 6.73 to 10	8.39 7.5 to 9.3	*p* = 0.00025
Time Interval Between Injections (weeks) 95% C.I.		5.7 4.2 to 7.3	8.5 5.3 to 11.7	10.7 2.9 to 18.5	8.8 5.6 to 11.9	*p* = 0.02
n	29	29	24	18	29	

BCVA = best corrected visual acuity; CI = Confidence interval; CST = central subfield thickness; TMV = total macular volume

### Previously treated CRVO

The average age of the previously treated patients was 71.6 (95% CI: 66.6 to 76.6) years of age. Prior to the first faricimab injection, these eyes received a mean of 8.8 (95% CI: 4 to 13.5) injections including bevacizumab, ranibizumab, aflibercept, dexamethasone implant and brolucizumab. The average interval between the last anti-VEGF and the first faricimab injection was 8.4 (95% CI: 5.5 to 11.4) weeks. Eyes gained 9.8 letters (*p* = 0.52333, Friedman test for repeated measures) after a mean follow-up of 21.8 (95% CI:13.4 to 30.1) weeks and 3.4 injections with a last treatment interval of 11 weeks. Table 10 summarizes the visual, anatomic outcomes and the time interval between these injections of the previously treated CRVO eyes following 3 injections and at the last follow-up. Four eyes of previously treated eyes discontinued treatment with faricimab. Three eyes were switched back to aflibercept after the first faricimab injection. One eye switched back to aflibercept after the second faricimab injection. One of the eyes developed an acute anterior uveitis which was treated successfully with topical steroids after the first faricimab injection. She decided to switch back to aflibercept.

**Table 10:** Eyes with previously treated central retinal vein occlusion

	Baseline	1 Injection	2 Injections	3 Injections	Last Visit	
BCVA logMAR (Snellen) 95% C.I.	1.02 (20/209) 0.82 to 1.22	0.96 (20/182) 0.67 to 1.25	0.82 (20/132) 0.59 to 1.06	0.69 (20/98) 0.28 to 1.11	0.91 (20/162) 0.67 to 1.14	
Cumulative Letter Gain 95% C.I.		3.9 -0.7 to 8.6	9.5 -4.8 to 23.8	18.7 -1.9 to 39.3	9.8 -0.2 to 19.8	
CST (µm) 95% C.I.	498 401 to 595	380 281 to 478	296 234 to 358	380 247 to 320	347 266 to 428	
TMV (mm3) 95% C.I.	10.9 9.70 to 12.16	9.35 7.97 to 10.74	8.64 7.66 to 9.61	8.85 7.74 to 9.97	9.29 8.05 to 10.5	
Time Interval Between Injections (weeks) 95% C.I.	8.4 5.5 to 11.4	9.6 6.6 to 12.6	9.7 5.2 to 14.3	9.5 3.5 to 15.5	11 7.5 to 14.5	
n	23	23	18	15	23	

### BRVO

A total of 20 eyes were treated for BRVO. All of these were previously treated eyes that were switched to faricimab because of a poor response with the prior treatment. The average age of the previously treated patients was 68.2 (95% CI: 61 to 75.4) years of age. Prior to the first faricimab injection, these eyes received a mean of 10.7 (95% CI: 3.2 to 18.2) injections including bevacizumab, ranibizumab, aflibercept, brolucizumab and the dexamethasone implant. The average interval between the last anti-VEGF and the first faricimab injection was 9.3 (95% CI: 5.6 to 13) weeks. Patients had a mean total follow-up of 28.1 (95% CI: 15.3 to 40.8) weeks. During this time the eyes achieved a mean gain of 5.4 letters (*p* = 0.00862, Friedman test for repeated measures) after 3.5 injections and a last treatment interval of 11.1 weeks. Table 11 summarizes the visual, anatomic outcomes and the time interval between these injections following 3 injections and at the last follow-up. None of the eyes were switched to an alternative treatment.

**Table 11:** Eyes with previously treated macular edema secondary to branch retinal vein occlusion

	Baseline	1 Injection	2 Injections	3 Injections	Last Visit	*P* (Friedman Test for Repeated Measures)
BCVA logMAR (Snellen) 95% C.I.	0.43 (20/54) 0.32 to 0.55	0.29 (20/39) 0.17 to 0.42	0.38 (20/48) 0.20 to 0.55	0.54 (20/69) 0.23 to 0.85	0.34 (20/44) 0.17 to 0.51	*p* = 0.00862
Cumulative Letter Gain 95% C.I.		7 3.6 to 10.4	5.9 0.5 to 10.8	-1.9 -19.4 to 15.7	5.4 -3 to 13.8	*p* = 0.00862
CST (µm) 95% C.I.	351 281 to 421	282 252 t0 312	275 234 to 317	277 202 to 352	274 236 to 312	*p* = 0.01753
TMV (mm^3^) 95% C.I.	8.98 7.77 to 10.20	7.78 6.88 to 8.69	8.16 6.73 to 9.58	8.26 6.15 to 10.37	7.80 6.80 to 8.81	*p* = 0.00032
Time Interval Between Injections (weeks) 95% C.I.	9.3 5.6 to 13	6.09 3.68 to 8.5	11.4 7 to 15.8	14.7 5.9 to 23.4	11.1 6.6 to 15.6	*p* = 0.007
n	20	20	15	10	20	

BCVA = best corrected visual acuity; CI = Confidence interval; CST = central subfield thickness; TMV = total macular volume

### Other conditions

A total of 69 eyes were treated for other conditions including CNV secondary to CSCR (16 eyes), PCV (13 eyes), myopic CNV (17 eyes), pachychoroid neovasculopathy (4 eyes), CME secondary to retinitis pigmentosa (4 eyes), CNV secondary to angioid streaks (1 eye), NV secondary to uveitis (2 eyes), idiopathic CNV (2 eye), CME secondary to macular telangiectasia 1 (1 eye), pseudophakic CME (1 eye), CME secondary to a ruptured macroaneurysm (1 eye) and diabetic retinopathy/PDR (3 eyes). Due to the heterogeneity of these conditions and the small numbers we will not report the functional or anatomic outcomes of these patients in this report. We will however report the adverse events observed in this cohort of patients.

### Adverse events

In general faricimab was well tolerated. There were 3 eyes that developed anterior uveitis that responded to topical steroids. There were no cases of endophthalmitis or occlusive retinal vasculitis. One patient with treatment naive DME who was being treated bilaterally died 6 months after the last injection. The treating physician did not think that the death was related to the faricimab treatment.

## Discussion

Since their introduction two decades ago, several randomized clinical trials have shown the benefits of several VEGF inhibitors in the treatment of NV-AMD, DME and CME secondary to RVO [13–19]. However several studies report that patients treated in routine clinical practice receive fewer anti-VEGF injections and consequently fall short of the full potential of such therapy because they were undertreated. These patients achieve worse visual outcomes than those in randomized clinical trials. In order to obtain the maximum benefit of anti-VEGF therapy, patients need chronic monitoring and regular injections. Many patients can’t keep up with the burdensome monitoring and treatment [20, 21]. 

The Pan American Collaborative Retina Study Group reported the long term outcomes of patients with DME, CME secondary to RVO and NV-AMD treated with intravitreal bevacizumab during routine clinical practice in Latin America. The vast majority of patients were undertreated and did not maintain their initial visual acuity gains [5–7]. The AQUILA study reported the outcomes of intravitreal aflibercept 2 mg in eyes with DME and NV-AMD during routine clinical practice in Latin America [22, 23]. At 12 months of follow-up, the mean number of injections received varied anywhere from 3.7 to 5.2. Despite this poor adherence to the recommended doses leading to undertreatment, there was a mean gain of 8, 4.6, 5.2 and 3.1 letters in treatment naive DME, previously treated DME, treatment naive NV-AMD and previously treated NV-AMD eyes respectively. These studies serve as an illustration that barriers to optimal patient care exist in Latin America. These include patients not receiving the label-prescribed number of anti-VEGF injections, limited access to ophthalmology specialists, and lack of awareness of retinal diseases in general [24]. Access and adherence to treatment may be influenced by socioeconomic factors. Inadequate health insurance may limit the number of injections a patient may receive and their access to ophthalmology specialists [25]. In addition to these difficulties in optimizing patient care, significant risk factors for patient non-adherence and non-persistence to anti-VEGF treatment have been identified. These risk factors further impair the achievement of efficacy outcomes from randomized controlled trials in routine clinical practice [26, 27]. 

One strategy to improve the outcomes is to target new pathways other than VEGF. One such pathway is the angiopoietin/**t**yrosine kinase with **i**mmunoglobulin and **e**pidermal growth factor homology domains (Ang/Tie) pathway. The Ang-Tie signaling pathway is a key regulator of inflammation and vascular homeostasis in physiological angiogenesis, developmental angiogenesis and pathological angiogenesis. Tie2 is a tyrosine kinase receptor expressed by endothelial cells. Quiescent and mature vessels are maintained by Ang1, the natural ligand of Tie2. Activation of Tie2 by Ang1 reduces vascular permeability by strengthening tight junctions between endothelial cells. Ang2 drives vascular instability and amplifies the effects of VEGF-A. It destabilizes the vasculature and acts in concert with VEGF to facilitate endothelial cell migration and proliferation. Several retinal diseases including NV-AMD, RVO and DME all manifest Ang-2 upregulation [8, 28–32]. 

Faricimab is a bi-specific monoclonal antibody that targets both Ang-2 and VEGF. The TENAYA and LUCERNE were randomized, double masked, multicenter phase 3 clinical trials that compared the efficacy and safety of faricimab to aflibercept (2 mg) in patients with treatment-naive NV-AMD. Faricimab was shown to be non-inferior to aflibercept in the treatment of NV-AMD. However only 8–11% of participants in the TENAYA and LUCERNE trials were Hispanic or Latino [33]. The YOSEMITE and RHINE studies were multicenter, double masked, randomized non-inferiority phase 3 clinical trials that compared faricimab to aflibercept in eyes with center involved DME. Previously treated eyes were capped at 25% of the enrollment. Hispanic or Latino enrollment comprised 12 and 21% respectively.[34, 35], The BALATON (18% Hispanic or Latino enrollment) and COMINO (19% Hispanic or Latino enrollment) studies were multicenter, double masked, randomized, non-inferiority phase 3 clinical trials that demonstrated that treatment with faricimab led to visual acuity outcomes at 6 months that were non-inferior to treatment with aflibercept in treatment naive eyes with CME secondary to branch retinal vein occlusion (BRVO) and hemiretinal or central retinal vein occlusion (CRVO) respectively. When compared to aflibercept treated eyes, more eyes in the faricimab treated group achieved absence of fluorescein angiographic macular leakage [12]. Macular leakage has been shown to be a biomarker of vascular stability [36]. In all the above studies, faricimab demonstrated non-inferior vision gains, improved anatomic outcomes and a potential for extended dosing when compared to aflibercept [10–12]. 

Real-world evidence is required to fully understand the effectiveness of faricimab in routine clinical practice, and could provide useful insights into current opportunities for clinical practice optimization. Data from routine clinical practice is critical for assessing outcomes across a more diverse patient population. The impact of ethnic and genetic variations on treatment responses and outcomes needs to be studied further.

Since the approval by different regulatory agencies throughout the world, several studies have reported on the outcomes of eyes treated with faricimab during routine clinical practice. All of these studies have been conducted across the USA, Europe, Australia and Asia but none in Latin America. The vast majority of these reports have been on previously treated eyes with NV-AMD with some studies on DME [37]. 

A few studies report on the outcomes of treatment naive NV-AMD eyes [38–45]. In general, treatment naive NV-AMD eyes respond well to faricimab both in anatomic and functional terms. In the TRUCKEE study, 39 eyes had a mean improvement in BCVA of + 4.9 letters following the first injection. After the third injection, the improvement in 13 eyes was of 8.1 letters [38]. In another retrospective study from Wales, 66 eyes underwent 3 monthly loading doses of faricimab and then were subjected to a treat and extend protocol depending on the outcomes after the third injection. Treated eyes experienced a mean improvement of 2.5 letters in BCVA after the first injection which improved to 6 letters after the third dose [43]. Three retrospective Japanese studies with 40, 63 and 47 eyes respectively reported that following a loading dose of 3 consecutive monthly injections of faricimab, eyes with treatment naive NV-AMD developed a dry macula in 79.5%, 82% and 72% of eyes. Furthermore complete regression of polypoidal lesions was seen in 61.1% and 52% of eyes with PCV. The subfoveal choroidal thickness was also significantly decreased after faricimab treatment. In these studies the BCVA improved significantly [44, 45]. Our series also compares favorably with a gain of 9.1 letters over a 27 week follow-up obtained after a mean of 4 faricimab injections.

There are many more studies reporting on previously treated NV-AMD eyes [37]. In these eyes faricimab may be a better macular drying agent than aflibercept but the BCVA remains for the most part unchanged. In some eyes the BCVA may improve but multiple monthly doses may be required to obtain the maximum benefit from faricimab [37]. Furthermore switching to faricimab from other anti-VEGF drugs may extend the treatment interval [46–48]. The TRUCKEE study from the USA demonstrated that a single faricimab injection in 337 eyes previously treated with aflibercept, led to an improvement in BCVA of + 0.7 letter from baseline. After 3 injections the improvement was of + 2.7 letters [38]. The FARETINA-AMD study includes over 21,000 patients from the American Academy of Ophthalmology’s IRIS registry that initiated faricimab treatment. In this cohort the BCVA did not improve after 4 faricimab injections despite anatomic improvements [49]. A retrospective study from Wills studied 218 eyes that were switched to faricimab. This cohort of eyes received a mean of 34.2 anti-VEGF injections prior to the faricimab switch. They all were treated with at least 4 consecutive monthly intravitreal faricimab injections. The anatomic outcomes improved whereas the visual acuity was maintained [50]. A retrospective study from the Cole Eye Institute reported that 126 eyes were switched to faricimab [51]. Most of these patients were being treated with aflibercept (87%) with a mean treatment interval of 5.6 weeks. Switched eyes had at least 3 intravitreal faricimab injections. After a mean follow-up of 24.3 wks, the mean visual acuity and the mean interval time between injections did not change. The retinal anatomy improved following the switch as evidenced by the reduction in PED height and reduction in CST. Eleven (8.7%) eyes were switched back to their previous anti-VEGF drug. These included 2 eyes from one patient that developed intraocular inflammation [51]. In a small study of 25 eyes with persistent exudative activity despite intravitreal aflibercept given every 8 weeks, a single faricimab injection was able to reduce or completely dry out the macula in half of the cohort [52]. Another retrospective study from the USA reported that in 190 eyes with previously treated NV-AMD that were treated with at least 3 faricimab injections, patients received a mean of 7 faricimab injections and had a mean follow-up of 35 weeks. The mean dosing interval between the last two consecutive faricimab injections (7.6 weeks) was significantly longer than that for ranibizumab (5.2 weeks) and aflibercept (5.6 weeks). The mean BCVA improved from 0.33 to 0.27 logMAR and the mean CST also improved from 313 μm to 288 μm.[53] In an Australian study, thirty one eyes with active MNV despite being treated with 4–6 weekly aflibercept, ranibizumab or brolucizumab were switched to faricimab [54]. All eyes were treated with at least 4 doses of faricimab and had at least 6 months of follow-up. Prior to the faricimab switch, these eyes had a median number of 41 anti-VEGF injections. At the 6 month follow-up, almost a third of patients remained at the same 4–6 weekly re-treatment interval with faricimab despite the switch to faricimab. The remaining 71% were able to increase the re-treatment interval by 3.5 weeks [54] A retrospective study from the UK assessed the outcomes in aflibercept resistant eyes that were switched to faricimab [55]. Our results compare favorably to the above studies with a mean improvement of 6.6 letters after a mean of 4.3 faricimab injections and a follow up of 41.7 weeks in eyes that were switched to extend the treatment interval. Similarly in eyes that were switched to faricimab due to a worsening NV-AMD, the cumulative letter gain ranged from 4.5 to 11.6 letters after 1 to 4 injections. These eyes had a mean follow-up of 28.6 weeks and received a mean of 3.8 faricimab injections.

The presence of fibrosis or atrophy was not taken into account in these studies. These may have played a role in the lack of improvement in visual outcomes in previously treated eyes. Our patients were probably less chronic as evidenced by the lower mean number of previous treatments (13 vs. 34 in the Wills study) compared to the other studies. Chronicity is associated with fibrosis and atrophy. The VOYAGER study is a phase IV study that will assess the effectiveness and safety of faricimab in patients with NV-AMD in routine clinical practice [56]. 

Short term outcomes of eyes with DME treated with faricimab during routine clinical practice have been reported in both previously treated and treatment naive eyes [37, 57–62]. A systematic literature review and network meta-analysis of 26 studies revealed that faricimab given on a treat and extend protocol was superior when compared to other drugs in decreasing retinal thickness [63]. In the FARETINA DME study, after a mean follow-up of 10 months, 851 treatment naive eyes gained 3.6 letters and a decrease of 52 μm in the CMT after the fourth faricimab injection [64]. Our results compare favorably with the above. After a mean follow-up of 25 weeks and 4.2 faricimab injections, we report a visual gain of 12.1 letters and a last treatment interval of 9.2 weeks in treatment naive DME eyes.

A recent systematic review and meta-analysis of 15 studies that included 629 eyes reported that eyes with persistent DME benefited from a switch to faricimab [65]. The FARETINA-DME study reported on over 6,200 previously treated eyes with DME from the AAO Iris registry. After 4 faricimab injections, there was a mean decrease in CMT of 34 μm and the BCVA remained unchanged at 65 letters [64]. A small retrospective case controlled study of 51 eyes with persistent DME despite aflibercept treatment compared the anatomic and functional outcomes of eyes switched to faricimab to those who continued aflibercept. Patients that were switched to faricimab received 3 consecutive monthly injections whereas those who remained on aflibercept also received 3 consecutive monthly injections. One month after the last injection, 37.5% (9/24) of faricimab treated eyes and 3.7% (1/27) of aflibercept treated eyes had an OCT with no retinal fluid and a CMT ≤ 300 μm. There were 41.7% of faricimab treated eyes and 11.1% of aflibercept eyes that gained ≥ 2 lines of BCVA [58]. In another retrospective study, 51 eyes with recalcitrant DME despite aflibercept therapy were switched to faricimab. Faricimab treated patients underwent a loading dose of 3 consecutive monthly injections and were then assessed for retinal fluid. If there was no more retinal fluid, the eyes were treated on a treat and extend protocol. At the end of 12 months, 39.2% of eyes achieved a dry macula and a treatment interval ≥ 8 weeks. The average reduction in CMT and the average improvement in BCVA were statistically significant [57]. A retrospective study of 18 eyes that had residual IRF or SRF or had a treatment interval less than 8 weeks despite ranibizumab or aflibercept treatment were switched to as needed faricimab. At 4 months, the treatment interval was increased from 5.8 to 10.8 weeks following the switch to faricimab. In 44% of eyes the treatment interval was extended to ≥ 12 weeks. In 16.7% of eyes a monthly treatment interval was required after the switch. Despite the switch to faricimab, the BCVA and the CMT were not significantly different [59]. Another retrospective Japanese study reported on 21 eyes that had a minimum follow-up of one month following faricimab treatment. In this cohort there were 14 treatment naive eyes and 7 previously treated eyes. After a mean follow-up of 5.5 months, eyes received a mean of 1.6 faricimab injections. Six (29%) eyes (4 treatment naive and 2 previously treated) were switched to alternative treatments such as aflibercept, brolucizumab, sub-Tenon triamcinolone and pars plana vitrectomy for various reasons including lack or insufficient response to faricimab and faricimab side effects [61]. In our cohort we found that after a mean follow-up of 29 weeks and 3.9 injections, these eyes gained a mean of 6.8 letters with a last treatment interval of 10.2 weeks (Table).

Currently there is very limited experience with faricimab in eyes with CME secondary to RVO in routine clinical practice. In a small retrospective study, 19 eyes with a poor response to ranibizumab, aflibercept or the dexamethasone implant received a loading dose of four consecutive monthly injections of faricimab. There was a mean improvement in both visual acuity and anatomic outcomes. The main weakness of this series is that given the small numbers, CRVO and BRVO were lumped together [66]. In our series we reported significant improvements in both BCVA and CMT in treatment naive CRVO and previously treated BRVO eyes after treatment with faricimab. In contrast previously treated eyes with CME secondary to CRVO did not improve functionally but did show an anatomic improvement. We suspect that these eyes may have sufferered from macular ischemia although we did not have OCT angiography or fluorescein angiography to confim this hypothesis. Future studies should report BRVO and CRVO separately and use either OCT angiography and/or fluorescein angiography to assess for macular ischemia.

Most patients in our series did not receive the label-recommended loading doses with a mean of 2.9 to 4.2 injections of faricimab over a mean follow-up of 23–33 weeks. Despite this undertreatment, our study shows that, regardless of the indication, our visual results compare favorably with what has been reported in the literature with a mean gain of 6.8 to 22.6 letters depending on the underlying condition and whether or not they had been previously treated or not. Furthermore the last mean treatment interval varied from 8.8 to 10.5 weeks.

There are several limitations to the current study due to its retrospective nature including the use of non-standarized visual acuities, the lack of a standarized follow-up protocol, 6 the lack of a standard protocol for injections and the short follow-up. Due to the small numbers, eyes with macukar edema secondary to BRVO and CRVO were lumped together.

Despite these limitations this study represents the largest and the first study to assess the use of faricimab for different retinal conditions in routine clinical practice in Latin America. The early Latin American experience with intravitreal faricimab is promising. Reported adverse events were few and few eyes were switched back to aflibercept. Future reports from the FARI-LATAM study will provide longer term findings on the outcomes of faricimab in routine clinical practice.

## Data Availability

Data is provided within the manuscript.
